# Effect of Different Temperature-Controlled Ultrasound on the Physical and Functional Properties of Micellar Casein Concentrate

**DOI:** 10.3390/foods10112673

**Published:** 2021-11-03

**Authors:** Bong Song, Yumeng Zhang, Baojia Yang, Panpan Zhu, Xiaoyang Pang, Ning Xie, Shuwen Zhang, Jiaping Lv

**Affiliations:** Institute of Food Science and Technology, Chinese Academy of Agricultural Sciences, Beijing 100193, China; 82101192170@caas.cn (B.S.); 82101191117@caas.cn (Y.Z.); 82101205366@caas.cn (B.Y.); 82101195218@caas.cn (P.Z.); pangxiaoyang@caas.cn (X.P.); xiening@caas.cn (N.X.); lvjiaping@caas.cn (J.L.)

**Keywords:** ultrasound, micellar casein concentrate, temperature-controlled, emulsification

## Abstract

Micellar casein concentrate (MCC) is a novel dairy ingredient with high protein content. However, its poor functional properties impair its potential for further application, highlighting the importance of using innovative processing methods to produce modified MCC, such as ultrasound (US). This work investigated the impact of US on the physical and functional properties of MCC under temperature-controlled and -uncontrolled conditions for different time intervals. Under temperature-controlled ultrasound (TC-US) treatment, a reduction was found in the supernatant particle size of casein micelles. Soluble calcium content and hydrophobicity increased following ultrasound treatment at 20 °C, resulting in a remarkable improvement in emulsification. However, long-time ultrasonication led to an unstable state, causing the MCC solutions to show shear thinning behavior (pseudoplastic fluid). Compared with 50 °C temperature-controlled ultrasonication, ultrasonication at 20 °C had a greater influence on particle size, viscosity and hydrophobicity. These findings indicate that 20 °C TC-US could be a promising technology for the modification of MCC.

## 1. Introduction

Ultrasound (US) is one of the rapidly developing innovative and green techniques that offer great potential for implementation in the food industry. Compared with other techniques (microwaves, gamma radiation), sound waves are generally considered safe, non-toxic, and environmentally friendly, making the use of ultrasound highly advantageous [1,2]. Compared with high-frequency ultrasound (20–100 kHz), low-frequency ultrasound (2–10 kHz) has found application in a wide range of dairy research applications, such as inactivation of microorganisms, extraction of components from cells or tissues, acceleration enzymatic activity, and dissolution of large particles [3,4,5,6].

Milk, a rich source of nutrients, can be fractionated into a wide range of components for use in food and beverages [7,8]. Micellar casein concentrate (MCC) is a novel ingredient with high casein content and a minor amount of lactose and minerals that is manufactured by microfiltration (MF) of skim milk, and is commercially available as a liquid, concentrate, or dried powder containing ≥8, ≥22, and ≥80% total protein, respectively [9,10]. MCC is widely used in cheese and ice cream production. However, due to its relatively poor solubility and emulsification, a large content of emulsifying salt (ES) needs to be used to achieve uniformity. Excessive addition of ES always leads to a decrease in thermal stability and hardness, highlighting the need to produce modified MCC. The current research on the effects of US on MCC is mainly focused on the modification of redissolved MCC powder. Some studies have shown that ultrasound could improve its solubility and intestinal digestibility [11,12]. These ultrasound treatments have often been performed for short periods (<10 min) without temperature control or for temperature controlled for long periods at low temperatures (<10 °C) on a laboratory scale. However, few studies have been published about the effect of US on MCC at different medium temperatures.

Medium temperature is also a key factor that can affect the generation of bubbles and the cavitation effect. More bubbles will be generated at higher temperatures because of the decrease in medium viscosity and tensile strength, further increasing the cavitation effect [13]. However, whey protein will be denatured if the temperature reaches above 65 °C. Hence, it is necessary to choose the appropriate medium temperature for US treatment. In this research, room temperature (20 °C) and medium-high temperature (50 °C) were chosen. The objective was to examine the effect of ultrasound under uncontrolled and controlled temperature on the physical and functional properties of MCC solutions, with a focus on the changes of particle size, viscosity, emulsifying activity index and fluid behavior.

## 2. Materials and Methods

### 2.1. Materials

Raw cow’s milk was obtained from Sanyuan First Farm (Beijing, China) and separated at 63 °C to remove fat (FT15-A, Armfield Limited, Ringwood, UK). Then, the skim milk was pasteurized in a plate heater at 72 °C for 15 s [14]. After pasteurization, the micellar casein concentrate was obtained according to our previous work [15,16] by using a 3-stage pilot-scale microfiltration system equipped with ceramic membrane with an average pore size of 100 nm (DONG QIANG MEMBRANE, Zibo, China). During the whole process, the concentration factor of the retentate was 3 and the transmembrane pressure was controlled at 0.5 MPa. Finally, the micellar casein concentrate contained 7.52% (*w*/*w*) casein, 0.92% (*w*/*w*) serum protein, 0.44% (*w*/*w*) lactose, and 0.29% (*w*/*w*) fat.

### 2.2. Pilot-Scale Ultrasound System

**Figure 1:**
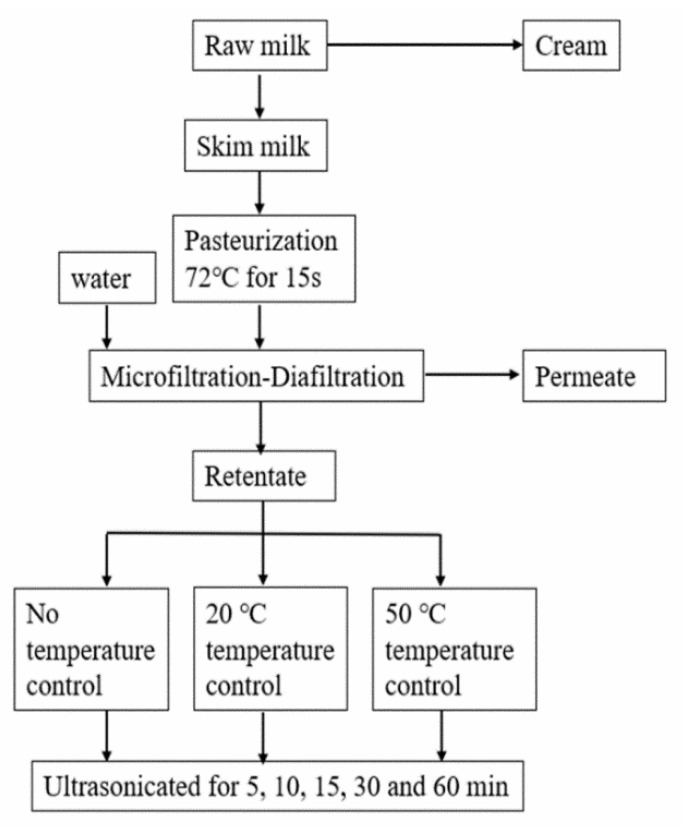
MCC production. After ultrafiltration and diafiltration, the retentates were treated for different US time and temperature.

The experimental scheme used is depicted in Figure 1. Micellar casein concentrate was sonicated in a 7.5 L metal tank equipped with a heat exchanger and cooling water jacket, using a 20 kHz, 4000 W ultrasonic horn (70 mm diameter, Hongxianglong Biotec, HXL-4000, Beijing, China) at an amplitude of 50% for 5, 10, 15, 30 and 60 min. The power output was determined to be 453 W by calorimetry and the energy density was calculated using the Eq as follows [17]:

Energy density = (P × t)/v(1)

where P represents the output power (W), t is the time (s), and v the volume of samples (mL). On the basis of the calculation, the energy densities at 5, 10, 15, 30 and 60 min of residence time were 18, 36, 54, 108 and 216 J/mL, respectively. For the temperature-controlled group, the MCC solution temperature during US treatment was maintained at 20 ± 2 °C and 50 ± 2 °C, respectively.

### 2.3. Average Particle Size

The MCC solution was divided into two parts; one part was used to measure the overall average particle size, the other part was centrifuged at 4000× *g* for 15 min and the upper liquid was taken to measure the particle size. A Malvern Master Sizer 3000 (Malvern Instruments, Worcester, UK) was used to measure the particle size with the refractive index of 1.58 at 25 °C [12]. Five measurements were obtained for each sample.

### 2.4. Zeta Potential Measurements

Zeta potential measurements were obtained using the Zetasizer (Nano series, Malvern Instruments, Worcester, UK) at 25 °C according to the method described by Nazari et al. [18].

### 2.5. pH and Viscosity Measurement

The pH of the untreated and US-treated solutions was measured using a pH meter (lab cHem, Australia) at 25 °C. Viscosity of the MCC solutions were measured using a Rheometer cylinder (Physica MCR 502, Anton Paar Company, Melbourne, Austria) operated using Rheoplus software. Measurements were taken using a 25 mm measuring plate at 25 °C between the shear rates of 0.1 and 200 s^−1^ for 40 min. Shear (apparent) viscosity was recorded as a function of shear rate and fitted according to the power–law equation: τ = K γ^n^ [19], where τ is the sheer stress, γ is the sheer rate, K and n represent the consistency index (N s^n^/m^2^) and flow behavior index, respectively.

### 2.6. Soluble Ca Measurement and SDS-PAGE

Aliquots (10 mL) of the untreated, ultrasonic-treated MCC samples were transferred into centrifuge tubes, then ultracentrifuged at 90,000× *g* for 2 h using a P40ST rotor in a L-80XP Ultracentrifuge (Hitachi WX, Tokyo, Japan), and the supernatants were carefully pipetted. The soluble Ca and overall Ca content were determined with an inductively coupled plasma mass spectrophotometer (Agilent, Santa Clara, CA, USA) according to the method described by Nassar et al. [20]. The content of micellar calcium was the difference between total calcium and soluble calcium content. SDS–PAGE was performed using a 4% stacking gel and a 12% resolving gel on Mini-Protein Tetra System (Bio-Rad Company, Oakland, CA, USA) and gel documentation system (Clinx Science Instruments Co, Ltd., Shanghai, China) according to the method of Yanjun et al. [21].

### 2.7. Emulsion Activity Index (EAI) and Emulsion Stability (ES)

The emulsion activity index (EAI) and emulsion stability (ESI) were measured by the absorbance at 500 nm using a spectrophotometer (Gipp, UV-1900PCS, Shanghai, China) according to the method described by Zhang, Pang, Lu, Liu, Zhang and Lv [11]. However, the preparation for the emulsion was modified as follows: ten-milliliter portions of MCC solution were diluted to 40% (*w*/*w*) with ultrapure water; then, the commercial soybean oil was added at the ratio of 1:3 and mixed. A rotary homogenizer set for 1.5 min at 9000 rpm (T25, IKA Inc., Staufen, Germany) was used for the preparation of emulsions. The emulsifying activity index (EAI) and emulsion stability (ESI) were calculated as follows [22]:

EAI = (2 × 2.303 × D × A_0_)/(10000 × C × θ) (2)

ES = A_0_/(A_0_ − A_20_) (3)

where D is the dilution ratio, A_0_ is the absorbance of emulsion at 0 min, A_20_ is the absorbance of emulsion formed after 20 min, c is the protein concentration, and θ is the volume concentration of soybean oil.

### 2.8. Surface Hydrophobicity and Intrinsic Fluorescence Spectroscopy

Hydrophobicity and intrinsic fluorescence spectroscopy of the MCC solutions were measured as described by Zhong and Xiong [23], with some modifications. A series of protein concentrations (0.005–0.25%) (*w*/*v*) from each MCC solution were prepared using 10 mM phosphate buffer at pH 7.2. Then, 30 μL of ANS-NA (sodium-8-anilino-1- naphthalene sulfonate) was added to 4 mL of diluted samples and mixed. The relative fluorescence intensity (RFI) of the protein solutions was measured using a fluorescence spectrometer (F-2500, Hitachi, Ltd., Tokyo, Japan) at excitation and emission wavelengths of 390 nm and 470 nm. The hydrophobicity value was determined by a scatter plot of net RFI with protein concentration. For fluorescence spectroscopy, MCC samples were diluted to 0.05 mg/mL solutions. The excitation wavelength was set at 280 nm and the emission spectra were measured from 300 to 450 nm at a scanning speed of 1500 nm/min [24].

### 2.9. Statistical Treatment

All data are expressed as mean ± standard deviation (SD) from at least three independent trials. Statistical analysis of the experimental data was performed by analysis of variance (ANOVA), and the significance of the difference between means was determined by Duncan’s multiple range test (*p* < 0.05).

## 3. Results and Discussion

### 3.1. Average Particle Size

**Figure 2:**
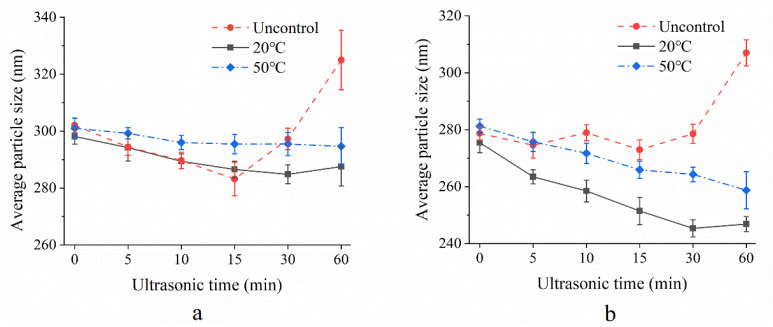
Overall average particle size (**a**) and supernatant average particle size (**b**) under uncontrolled, 20 °C and 50 °C temperature-controlled ultrasonication for different time intervals (*n* = 3 or 4): uncontrol (●), 20 °C temperature control (■), 50 °C temperature control (◆).

Figure 2a shows the overall average particle size of US-treated and untreated MCC samples as a function of US time under controlled (20 °C, 50 °C) or uncontrolled temperature conditions. The size of the soluble particles exhibited a slight decreasing trend from 0 min up to 30 min (energy density: 108 J/mL) under controlled temperature conditions. This result was in agreement with Shanmugam et al. [25], who reported 20 kHz ultrasound at 41 W could slightly reduce the particle size of pasteurized homogenized skim milk [26]. In our experiment, a maximum reduction in size occurred during the first 15 min (energy density: 54 J/mL) under uncontrolled temperature (about 15 nm smaller than initial solution), but US for longer than 30 min resulted in a large and progressive increase in the particle size. This phenomenon might be related to the denatured serum protein aggregation caused by high temperature in long ultrasonic time [27]. Under US treatment without temperature control, the temperature of the samples would achieve over 80 °C, resulting in denaturation of serum proteins. On this occasion, the levels of β-lactoglobulin and α-lactalbumin in serum phase decreased with prolonged US time, while the levels of κ-casein increased [25]. These newly formed denatured/aggregated soluble whey proteins would interact with κ-caseins present on the surface of the casein micelles or form aggregates in the serum phase, further exhibiting an increase in particle size.

When the MCC were centrifuged at 4000× *g* for 15 min, large micellar casein would be sedimented while native serum proteins, small micellar caseins, free caseins and serum protein aggregates still existed in the supernatant. Figure 2b showed the average particle size of MCC supernatants soluble particles as a function of sonication time under controlled or uncontrolled temperature. Except for uncontrolled temperature US treated MCC samples, the supernatant average particle size exhibited a significant (*p <* 0.05) downward trend with prolonged US time at 20 °C and 50 °C so that the size was roughly 20 nm and 40 nm smaller than the initial size. The decrease of the supernatant particle size could be due to the breakup of aggregated whey protein, whey protein associated with CN micelles that may have formed during pasteurization and small caseins which were dissociated from large micellar caseins. Furthermore, the decrease observed in the average particle size of supernatants under 20 °C temperature-controlled treatment was significantly higher in comparison to the size of uncontrolled temperature and 50 °C temperature-controlled samples.

**Figure 3:**
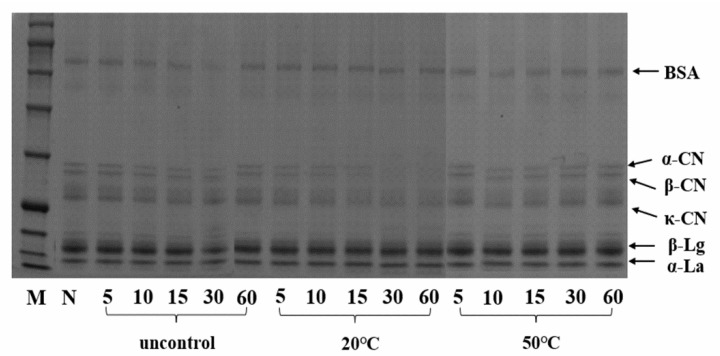
SDS-PAGE profiles of MCC supernatant (90,000 g for 2 h) treated by US without temperature control or US combined with temperature control at different processing times (5, 10, 15, 30 and 60 min) and controlled temperatures (20 °C, 50 °C). M: marker, N: native (untreated US) sample.

Apart from the dissociation of micelles, it was interesting to note that reaggregation of free caseins occurred during ultrasound treatment. After centrifuged at 90,000× *g* for 2 h, the supernatant was mainly composed of serum protein and free caseins. As could been seen in Figure 3, the bands of free α-caseins and β-caseins present in soluble supernatant were lighter at 30 and 60 min under 20 °C temperature-controlled US treatment, implying some free caseins were precipitated by centrifugation. A possible explanation for this observation may be due to the reaggregation of the caseins with casein micelles caused by hydrophobic interaction and sheer forces of acoustic cavitation. The turbulent conditions generated by the ultrasound treatment have also been shown to increase particle mobility and promote the formation of aggregates.

### 3.2. pH and Calcium Content

The pH and soluble calcium content could be used to indicate the integrity of casein micelles [28].

**Figure 4:**
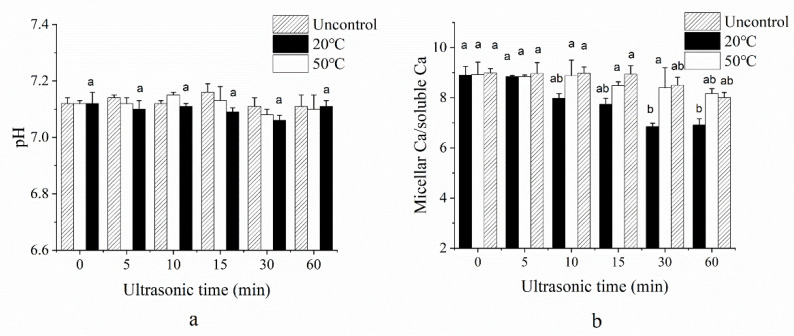
Mean (*n* = 4) pH value (**a**) and the ratio of micellar Ca and soluble Ca (**b**) of MCC retentate under uncontrolled, 20 °C and 50 °C temperature-controlled ultrasonication for different time intervals. Means across all samples tested with different letters (a,b) differ significantly (*p* < 0.05).

Figure 4a,b show the effect of sonication on the calcium distribution and solution pH, respectively. Although pH value had a tendency to decrease from 0 min up to 30 min under 20 °C and uncontrolled temperature samples, no significant difference was observed. Some researchers believed US could alter the pH of a solution because of the formation of small amounts of nitric acid, which were generated by oxygen and nitrogen reaction under heat zone from the cavitation effect [29]. However, some studies have indicated that ultrasound might disrupt the natural mineral equilibrium present in milk, further affecting the pH value [30]. As can be seen from Figure 4b, there was a significant increase (*p <* 0.05) in soluble calcium content in long time US treatment, especially at 30 and 60 min under 20 °C. The soluble calcium content rose from 320 to 384 ppm, indicating ultrasound changed the structure of micellar casein and caused the micellar calcium transferred to the serum phase. In addition, no significant difference was observed under 50 °C temperature-controlled US treated samples. This phenomenon could be ascribed to the high vapor pressure at high temperatures, which impairs the bubble generation and weakens the cavitation effect. The effect of ultrasound on calcium balance may be related to pressure effect caused by cavitation. High pressure probably induces disruption of ionic interactions between caseins and inorganic constituents, with a resulting release of calcium from the micelles to the soluble phase, but the exact mechanism of micelle dissociation on US treatment remained unclear.

**Figure 5:**
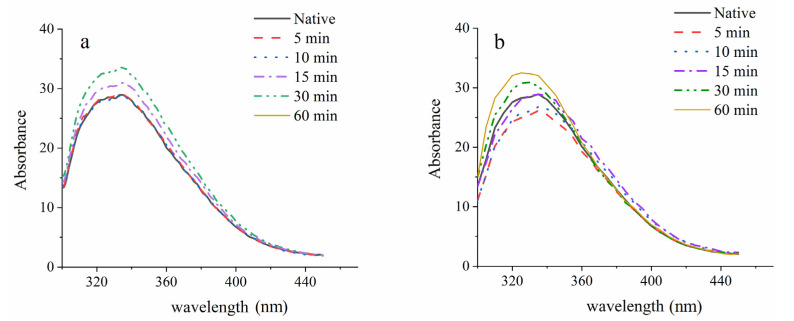
Fluorescence spectroscopy of micellar casein concentrate treated by ultrasound with 20 °C (**a**) and 50 °C (**b**) temperature control.

In milk protein systems, the removal of calcium ions has often been accompanied by protein structural changes. As can be seen from Figure 5, the fluorescence intensity under 20 °C and 50 °C US treated samples is changed, indicating the quaternary structure of the casein was disrupted. Furthermore, US treatment always occurs concurrently with secondary structural rearrangements and partial unfolding into more intensive structure. Hong Bui et al. [31] applied second derivative infrared spectroscopy to analyze the influence of ultrasound on treated milk systems at different ratios of caseins and serum proteins, and found prolonged sonication resulted in the decreased α-helix/large loop, and increased intermolecular β-sheet and random coil. This series structural and functional changes of MCC would heavily affect its application.

### 3.3. Rheological Properties

**Figure 6:**
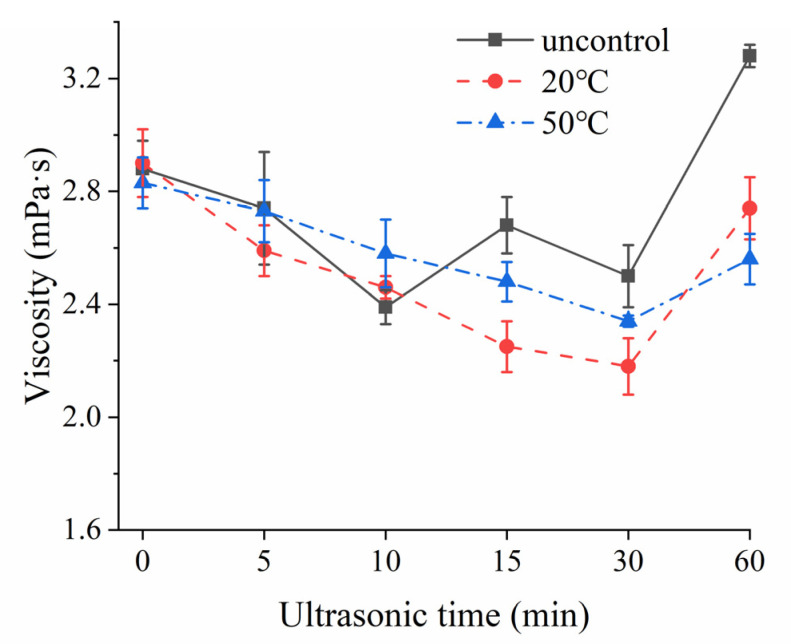
Mean (*n* = 4) viscosity of MCC samples at uncontrolled, 20 °C and 50 °C temperature-controlled ultrasonication for different time intervals: uncontrol (■), 20 °C temperature control (●), 50 °C temperature control (▲).

The viscosity value for each MCC sample was recorded at the shear rate of 200 s^−1^, as presented in Figure 6.

Except for uncontrolled temperature US treated group, the viscosity of MCC samples displayed a decreasing trend with prolonged sonication time (0–30 min) at 20 and 50 °C. Moreover, 20 °C temperature-controlled treatment showed a higher impact on viscosity reduction than that of observed at 50 °C. This result could be attributed to the particle size and zeta potential (Table 1).

The average particle size of the supernatant decreased with US time under 20 and 50 °C. Due to the decreased particles, the hydrodynamic diameter, molecular mobility and internal friction resistance was reduced, resulting in decreased viscosity. Similar results were obtained by the work of Deshpande and Walsh [32], who reported application of low-frequency ultrasound could reduce the viscosity of the reconstituted skim milk (rSM) and reconstituted milk protein concentrate (rMPC). However, in the work of Lo et al. [33], they reported that the application of low-frequency ultrasound had no effect on the viscosity of the sodium caseinate suspensions. This difference was primarily due to the absence of large aggregates in reconstituted sodium caseinate suspensions. The overall effect of low-frequency sonication was largely insignificant when the casein proteins were not present in the micellar state.

**Figure 7:**
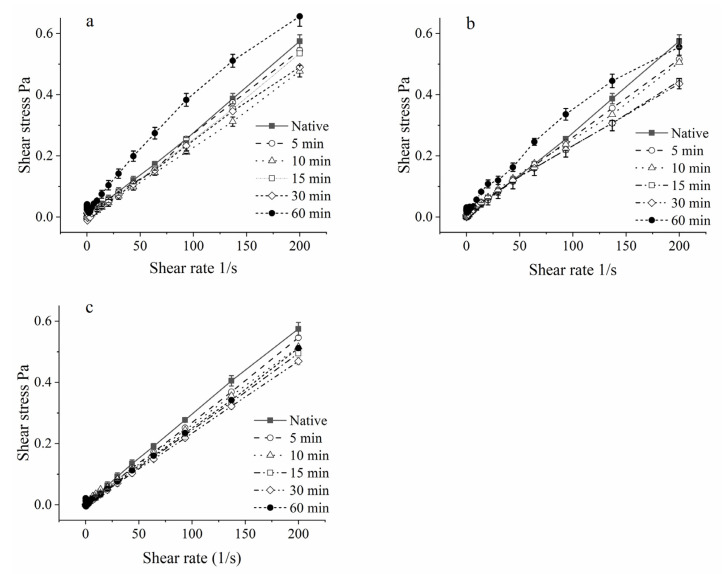
Mean (*n* = 3) viscosity of MCC samples at uncontrolled, 20 °C and 50 °C temperature-controlled ultrasonication for different time intervals: uncontrolled (**a**), 20 °C temperature-controlled (**b**), 50 °C temperature control (**c**).

When the MCC solution was ultrasonicated for 60 min, the zeta potential value was significantly lower than that of other samples, implying that the whole solution system was not stable and stronger intermolecular forces were formed between the particles. This would reduce the molecular mobility and made it hard to deform under external forces applied by the rotor, hence exhibiting an increase in apparent viscosity. Meanwhile, this unstable system had a huge influence on the state of the fluid movement, as shown in Figure 7. Except for 60 min ultrasound treatment under uncontrolled and 20 temperature-controlled groups, both shear stress and shear rate changed linearly at all temperature levels. Shear rate and shear stress were fitted according to the power-law equation: τ = K γ^n^ [34]. On the base of this calculation, the flow behavior index value of most US treated and untreated MCC samples was around 1, exhibting Newton fluid behavior [35]. However, the flow behavior index under 60 min for uncontrolled temperature and for 20 controlled temperature was 0.82 and 0.79, respectively, showing shear thinning behavior (pseudoplastic fluid). Thus, long-time ultrasound treatment could change the state of fluid movement. The formation of pseudoplastic fluid might be related to the entanglement networks caused by protein molecular chain [36]. There was less entanglement at high shear rates, while extensive entanglement at low shear rates [37].

### 3.4. The Emulsifying Activity Index (EAI) and Emulsion Stability (ESI)

Table 2 shows a significant improvement in EAI of MCC solutions under 20 temperature-controlled US treatment, in which the maximum EAI and ES were observed at 42.3 ± 1.9 and 87.1 ± 5.1%, respectively.

**Figure 8:**
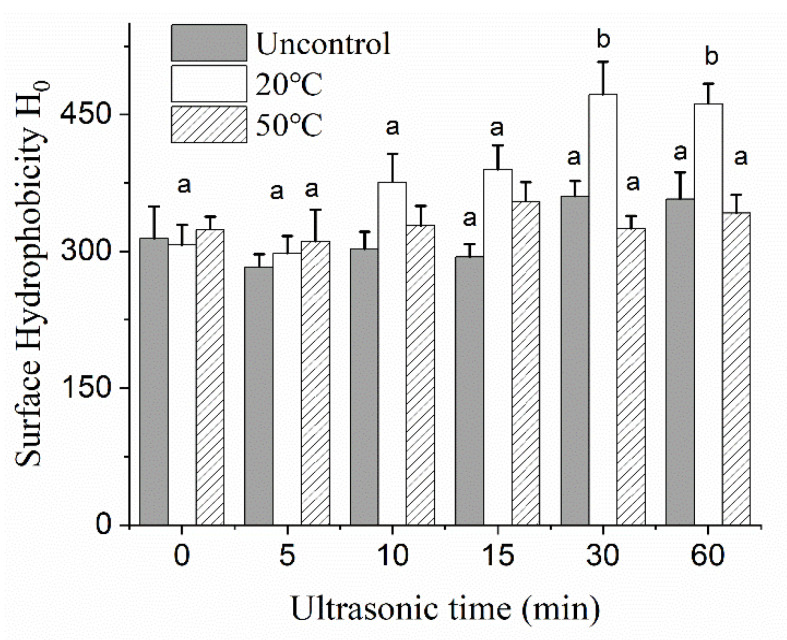
Surface hydrophobicity of MCC treated by ultrasonication without temperature control (dark column) and ultrasound combined with temperature control at different processing times (5, 10, 15, 30 and 60 min) and controlled temperatures (20 °C, 50 °C). Means across all samples tested with different letters (a,b) differ significantly (*p* < 0.05).

With increased US treated time from 0 to 15 min at 453 W output power at 20 °C, the EAI did not show significant changes, but an increase from 15 to 30 min led to significant improvement (*p <* 0.05) and a small decrease at 60 min. This result could be explained by the rearrangements structure of MCC, which increased the exposed internal hydrophobic groups on the surface of the protein, thereby reducing the interfacial tension and making it easier to be absorbed on the oil–water interface. However, excessive hydrophobicity might induce hydrophobic aggregation between proteins, further reduced the emulsification. The result in Figure 8 was in agreement with the observations of H_0_. In addition to the exposure of internal hydrophobic group, some studies revealed that the distribution of hydrophobic groups on the oil-water interface might also affect the emulsification. Amino acid sequence alignment and hydrophilic analysis revealed proteins with a well balance between hydrophobic and hydrophilic amino acid groups had high interfacial activity. In contrast, a long hydrophilic or hydrophobic fragment could adversely influence protein interfacial activity [38,39].

The EAI and H_0_ of the uncontrolled temperature group were observed to be slightly improved at 30 and 60 min. These results could be explained by the dissociation of partial micellar calcium caused by thermal effect, which promoted structure slight changes and the stretch of protein molecules, causing it to wrap around small oil droplets. However, the emulsification index at 60 min was lower than that of 20 °C temperature-control group, which might be attributed to the presence of large particle aggregates. Because of the heat treatment, a large number of protein aggregates would be formed after protein denaturation, making it unable to effectively cover the entire fat droplet, resulting in emulsion instability.

**Figure 9:**
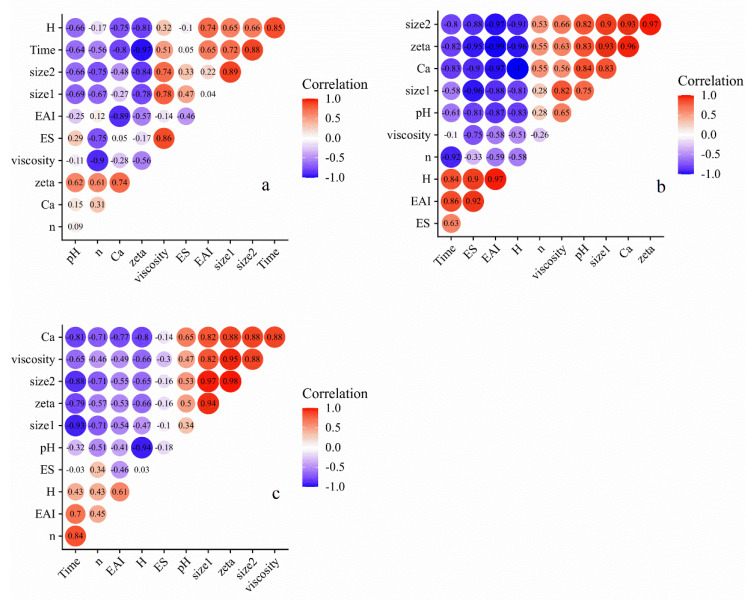
Correlation coefficient among different indexes of MCC samples treated by US at uncontrolled temperature (**a**), 20 °C (**b**) and 50 °C (**c**). Size1: Overall particle size. Size2: Supernatant particle size. Ca: Ratio of micellar calcium content to soluble calcium content. H: Surface hydrophobicity. n: Flow behavior index. EAI: Emulsion activity index. ES: Emulsion stability. Zeta: Zeta−potential.

It was interesting to note that no significant difference of EAI and surface hydrophobicity was observed at 50 °C US treatment. There were because of the high temperature of the system and the weakening of cavitation effect. Not only in terms of emulsification, but also with respect to particle size and viscosity, the effect of ultrasound at 50 °C seemed to be less significant than that of holding the temperature at 20 °C. The temperature of the solution was a key factor for the ultrasonic intensity. On the one hand, increasing the temperature during the ultrasonic process could reduce the energy threshold for generating cavitation bubbles, and the bursting of more cavitation bubbles generated more uniform and stronger shear force [40]. On the other hand, as the processing temperature further increased, the vapor pressure inside the cavitation bubbles would gradually increase and produced a certain buffer effect on the burst of cavitation bubbles, weaken the strength of ultrasonic cavitation effect [41]. In the work of Zhong and Xiong [23], they found changes in secondary structure of MPI were minimal at 30 and 50 °C but were significant at 70 °C. Furthermore, the dissociation of native components followed by reaggregation into soluble particles following ultrasound treatment at 70 °C resulted in remarkable improvements of protein solubility, clarity, and stability of the mung protein isolate suspensions. However, Ashraf et al. [42] compared the effect of thermo-sonication pretreatment on mung bean under various temperature. FTIR analysis revealed that marked spectral changes were noted after ultrasound treatment at 20 °C rather than at higher temperature. This difference was primarily due to the composition and solid content of the solution. As demonstrated in Figure 9, the correlation heat map showed that a huger number of dark areas was observed under 20 °C temperature-controlled US in comparison to those of at 50 °C and uncontrolled temperature, implying a closer connection among different indexes. In our experiment, the impact of cavitation effect at 20 °C was more intensive than that of compared to 50 °C.

## 4. Conclusions

The effect of temperature-controlled ultrasound on the physical and functional properties of micellar casein concentrate were studied on a pilot scale. The present study showed that 20 °C US treatment (20 kHz, 453 W and 50% amplitude) for 30 min significantly improved emulsifying activity index. This could help expand the application of MCC in food industry.

Although US could slightly dissociate casein micelles, aggregates could be formed after long-time US treatment, whether at or without temperature control. The former was mainly caused by hydrophobic interaction, while the latter was primarily attributed to the denatured serum protein. Prolonged US time resulted in the change of fluid state, causing it to display shear thickening behavior, indicating the unstable state of the solution system. Therefore, long-time (60 min) ultrasound should be avoided in industrial applications, because the unstable state of US treated samples would affect its subsequent processability and the final quality of the product.

Temperature control was crucial for the ultrasound effect on MCC solutions. Compared with 50 °C temperature-controlled sonication, 20 °C had a stronger influence on particle size, viscosity and hydrophobicity, which might imply a stronger cavitation effect.

Therefore, US treatment with temperature control can be used to improve MCC functional properties and make the modified protein as a potential ingredient in different food applications including cheese, cake and ice cream. However, further research on the application in the real food system is still necessary, and this is one of our next plans.

## Figures and Tables

**Table 1:** Effect of ultrasound treatment at different temperatures and time intervals on zeta-potential of micellar casein concentrate.

Temperature (°C)	Time (min)	End Temperature ^1^ (°C)	Energy Density (J/mL)	Zeta-Potential (mV)
Native ^2^	0	-	0	−30.2 ± −1.3 ^a^
uncontrol	5	32	18	−28.1 ± −1.1 ^a^
	10	39	36	−29.6 ± −1.7 ^a^
	15	47	54	−28.9 ± −1.2 ^a^
	30	62	108	−26.1 ± −0.6 ^ab^
	60	84	216	−22.5 ± −0.4 ^c^
20 °C	5	20	18	−28.5 ± −1.8 ^a^
	10	21	36	−25.3 ± −2.1 ^ab^
	15	20	54	−23.2 ± −2.1 ^bc^
	30	20	108	−21.4 ± −1.3 ^c^
	60	22	216	−21.2 ± −0.8 ^c^
50 °C	5	49	18	−28.8 ± −0.7 ^a^
	10	50	36	−26.6 ± −1.4 ^b^
	15	51	54	−25.2 ± −1.4 ^ab^
	30	52	108	−24.4 ± −0.3 ^b^
	60	52	216	−24.2 ± −0.3 ^b^

Values are means (*n* = 5); means in the same column not sharing a common superscript letter are significantly different (*p <* 0.05). ^1^ End temperature: the temperature of MCC after US-treated. ^2^ Native: none US treated mi cellar casein concentrate sample.

**Table 2:** Effect of ultrasound treatment at different temperatures and time intervals on emulsion activity index (EAI) and emulsion stability (ES) of micellar casein concentrate solutions.

Temperature (°C)	Time (min)	Energy Density (J/mL)	EAI (%)	ES (%)
native	0	0	28.6 ± 0.7 ^a^	74.5 ± 5.4 ^b^
uncontrol	5	18	28.8 ± 0.6 ^a^	73.9 ± 7.1 ^b^
	10	36	30.9 ± 0.8 ^ab^	65.6 ± 4.6 ^a^
	15	54	34.3 ± 1.3 ^b^	71.2 ± 5.3 ^b^
	30	108	37.1 ± 2.4 ^c^	66.2 ± 2.1 ^a^
	60	216	33.9 ± 2.0 ^b^	74.1 ± 2.0 ^b^
20 °C	5	18	30.8 ± 4.2 ^ab^	75.0 ± 4.5 ^b^
	10	36	34.4 ± 2.9 ^b^	82.2 ± 7.6 ^c^
	15	54	37.8 ± 0.9 ^c^	86.7 ± 6.8 ^c^
	30	108	42.3 ± 1.9 ^c^	87.1 ± 5.1 ^c^
	60	216	42.4 ± 2.5 ^c^	84.9 ± 1.7 ^c^
50 °C	5	18	26.9 ± 2.3 ^a^	78.6 ± 2.9 ^bc^
	10	36	27.5 ± 3.2 ^a^	75.4 ± 2.2 ^b^
	15	54	28.5 ± 1.2 ^a^	77.2 ± 1.9 ^bc^
	30	108	28.8 ± 1.9 ^a^	76.8 ± 3.6 ^b^
	60	216	29.4 ± 2.0 ^a^	75.9 ± 4.0 ^b^

Values are means (*n* = 4); means in the same column not sharing a common superscript letter are significantly different (*p* < 0.05). Native: none US treated micellar casein concentrate sample.

## Data Availability

The data presented in this study are available on request from the corresponding authors.
